# Chinese herbal medicines as a source of molecules with anti-enterovirus 71 activity

**DOI:** 10.1186/s13020-016-0074-0

**Published:** 2016-01-28

**Authors:** Mengjie Wang, Ling Tao, Hongxi Xu

**Affiliations:** School of Pharmacy, Shanghai University of Traditional Chinese Medicine, Shanghai, 201203 China; Engineering Research Center of Shanghai Colleges for TCM New Drug Discovery, Shanghai, 201203 China; Xinxiang Medical University, Jinsui Road 601, Xinxiang, Henan 453003 China

## Abstract

Enterovirus 71 (EV71) is one of the causative agents of hand, foot, and mouth disease (HFMD), which sometimes leads to severe neurological disease and death in the Asia–Pacific region. In Chinese medicine, HFMD is caused mainly by an accumulation of *damp*-*heat* and *toxicity* in the body. No effective drugs are currently available for the treatment and prevention of EV71 infection. This review summarizes the potential Chinese herbal extracts and isolated compounds with antiviral activity against EV71 and their clinical applications, especially those categorized as *heat*-*clearing* and *detoxifying*.

## Background

Enterovirus 71 (EV71) is a non-enveloped, positive-sense, single-stranded RNA virus that is 7.4 kb in length and belongs to the family* Picornaviridae* [1]. Infection with EV71 commonly causes mild hand, foot, and mouth disease (HFMD), which sometimes leads to serious neurological complications such as aseptic meningitis, brain stem encephalitis, pulmonary edema, and poliomyelitis-like paralysis, and eventually causes death especially in infants and children [2]. The potential fatal implications pose a great threat to infants and children under 5 years of age [3]. Since the first isolation of EV71 in the United States in 1969, there have been several outbreaks of EV71 in Bulgaria, Malaysia, Taiwan, and China that caused considerable levels of infection and mortality [4–7]. However, neither a precautionary vaccine nor a specific antiviral drug is available for the treatment of EV71 infection [6].

Interferons (IFNs) are a group of antiviral proteins (mainly glycoproteins) that regulate host cytokines and chemokines [8]. Infection with EV71 attenuates the IFN response, and reduces the antiviral effect of IFNs [9]. Although ribavirin, a nucleoside analog, has been clinically used as a broad-spectrum antiviral drug, treatment of EV71 infection by ribavirin has been unsatisfactory with considerable side effects in infants and children [10, 11]. Pleconaril, an antipicornavirus capsid-binding agent, shows moderate efficacy in anti-EV71 treatment, but cannot reduce the cytopathic effect (CPE) induced by some EV71 strains [12, 13]. Rupintrivir, an inhibitor of human rhinovirus (HRV) 3C protease, specifically binds to the 3C protease of EV71, inhibits the replication of EV71 in vitro, and strongly contains the spread of EV71 infection in vivo [14]. However, this drug has not yet been used for clinical treatment of EV71 infection.

In addition to synthetic compound design, Chinese herbal medicines (CHMs) contain a wide range of phytochemicals and comprise a potential source of anti-EV71 active agents [15]. According to Chinese medicine (CM) theory, diseases occur and develop through the effects of pathogenic factors on the human body that lead to an imbalance of *qi*, *xue*, *yin*, and *yang*, or *organs* and *meridians* in the body [16]. Herbs have different impacts on the human body to regulate *qi*, *xue*, *yin*, and *yang*, and balance the whole body. HFMD is caused mainly by an accumulation of *damp*-*heat* and *toxicity* in the body, and therefore its treatment may involve the usage of *heat*-*clearing* and *detoxifying* medicines. In this review, studies on CHM extracts and compounds with anti-EV71 activity are summarized, based on CHMs categorized into *heat*-*clearing* and *detoxifying* medicines. Future perspectives and challenges in anti-EV71 drug development involving herbal medicines are also discussed.

## Extracts with anti-EV71 activity

Extracts of CHMs have shown significant antiviral effects against EV71 with low EC_50_ values and high selective index (SI) values. Previous studies, formulae, and clinical experiences with CHMs and ethnomedicines revealed inhibitory effects on a range of viruses, such as influenza virus, hepatitis B virus (HBV), dengue virus, and coxsackie virus B3 (CVB3) [17–20]. Extracts of CHMs may be complementary to modern medicines. Extracts with anti-EV71 activity are summarized in Table 1.

**Table 1:** Anti-EV71 effects of extracts from Chinese medicines

Category	Plant source and reference	Fraction	Anti-EV71 effect	Mechanism
*Heat*-*clearing* and *detoxifying* medicine	*Houttuynia cordata* Thunb. (*Yu Xing Cao*) [27, 28]	Water extract	Reduces CPE (EC_50_ 125.92 μg/mL, SI 101.65), virus titre, plaque formation (EC_50_ 8.9–20.6 μg/mL, SI above 48), viral RNA production and 3A protein expression, inhibits EV71-induced apoptosis, prevents IκBα degradation, and down-regulates IL-6	Inhibits viral replication, and proinflammatory response
	*Paris polyphylla* Smith (*Qi Ye Yi Zhi Hua*) [32]	95 % ethanol extract	Reduces CPE and plaque formation (EC_50_ 78.46–125.00 μg/mL, SI 5.96-9.49), raises IL-6 level	Destroys virus
	*Kalanchoe gracilis* (*Deng Long Cao*) [36]	Water extract	Reduces CPE and plaque formation (EC_50_ 35.88 μg/mL, SI above 27), and virus yield, inhibits EV71-induced apoptosis, viral 2A protease activity, expression of IL-6 and RANTES, and reduces the viral load in intestine of suckling mice	Inhibits viral protease activity, viral RNA replication, and influences host cell factors
	*Kalanchoe gracilis (Deng Long Cao)* [37]	Ethyl acetate extract	Reduces CPE (EC_50_ 4.21 μg/mL, SI above 97) and plaque formation	Inhibits virus binding
	*Saururus chinensis* (Lour.) Baill (*San Bai Cao*) [38]	Water extract	Reduces CPE (EC_50_ 8.9 μg/mL) and virus titre	Inhibits activation of MEK1-ERK signalling pathway
	*Paulownia tomentosa* (*Pao Tong*) [40]	Methanol extract	Reduces CPE (EC_50_ 65 μg/mL)	Inhibits viral RNA replication
	*Phyllanthus urinaria* (*Zhen Zhu Cao*) [41]	Ethyl acetate, and butanol extracts	Reduces CPE	
Other Chinese medicine	*Salvia miltiorrhiza* (*Dan Shen*) [43]	Water extract	Reduces CPE (EC_50_ 0.742 mg/mL for SA1, 0.585 mg/mL for SA2), plaque formation, viral yield, and EV71-induced apoptosis	Inhibits viral RNA synthesis
	*Puerarla lobata* (*Ge Gen*) [48]	Water extract	Reduces CPE (EC_50_ 0.028 μg/mL, SI 107,000), and decreases IFN production	Inhibits viral attachment and penetration
	*Glycyrrhiza uralensis* (*Gan Cao*) [50]	Water extract	Reduces CPE (EC_50_ 0.056 μg/mL, SI 5000)	Prevents viral attachment and penetration
	*Ampelopsis brevipedunculata* Trautv (*Shan Pu Tao*) [51]	Acetone extract	Reduces CPE and plaque formation (EC_50_ 26.11 μg/mL, SI 5.56), and down-regulated IL-6, IL-1β, IL-8 levels	Prevents viral infection, inactivated virus, and inhibited viral replication
	*Daphne Genkwa* Sieb. et Zucc. (*Yuan Hua*) [59]	Water extract	Reduces CPE (EC_50_ 0.163–0.824 mg/mL, SI 1.752–8.859), virus yield, virus titre	Inhibits viral attachment and penetration
Chinese medicinal formulae	*Sheng*-*Ma*-*Ge*-*Gen*-*Tang* [61]	Water extract	Reduces CPE (EC_50_ 0.21 μg/mL, SI above 23809.52)	Inhibits viral attachment and penetration
	*GuiQi* Polysaccharides [62]	Water extract precipitated with ethanol	Reduces CPE (EC_50_ below 31.2 μg/mL)	Inhibits viral adsorption

### Heat-clearing and detoxifying medicines

*Heat*-*clearing* and *detoxifying* medicines comprise a variety of herbal medicines that can be used to treat heat-related syndromes such as high body temperature, thirst in the mouth and throat, constipation, inflammation, and pain [21].

*Houttuynia cordata* Thunb. (*Yu Xing Cao*) eliminated *heat* and *toxicity* in the human body and promoted urination [22]. The herb exhibited anti-inflammatory, anticancer, and antiobesity activities, and blocked infection of herpes simplex virus (HSV) [23–26]. In a screening of 22 CHMs, a water extract of *H. cordata* Thunb. inhibited the CPE and plaque formation induced by EV71 in Vero cells with an EC_50_ of 125.92 µg/mL [27]. The *H. cordata* Thunb. extract (125 µg/mL) lowered the 50 % viral RNA yield, reduced viral protein 3A expression, and inhibited EV71-induced apoptosis in comparison with the untreated group, and among five tested pure compounds extracted from *H. cordata* Thunb., chlorogenic acid showed a moderate anti-EV71 effect with an IC_50_ of 102.53 µg/mL. The water extract of *H. cordata* Thunb. had anti-EV71 activity against the Fuyang and BrCr strains in Vero cells with EC_50_ values of 8.9 and 20.6 µg/mL, respectively [28]. Incubation of the extract before or during inoculation significantly suppressed EV71 infection and also inhibited the CPE of coxsackievirus A16 (CVA16), another causative agent of HFMD. Pre-treatment with the extract prevented EV71-induced IκBα degradation and downregulated interleukin (IL)-6 gene expression.

*Paris polyphylla* Smith (*Qi Ye Yi Zhi Hua*) is used for the treatment of snake bites [29]. *P. polyphylla* Smith possessed anticancer activity, and saponins from the herb exhibited antifungal effects [30, 31]. The 95 % ethanol extract of *P. polyphylla* Smith showed antiviral activity against four strains of EV71 and CVB3 with EC_50_ values of 78.46–125.00 µg/mL [32]. The extract inhibited both viral replication and associated increases in IL-6 levels.

*Kalanchoe gracilis* (KGS) (*Deng Long Cao*) can be used to treat injuries, pain, inflammation, and fever because of its antioxidative, anti-inflammatory, analgesic, and anticancer activities [33–35]. The water extract of KGS leaves exerted antiviral effects against EV71 and CVA16 with EC_50_ values of 35.88 and 42.91 µg/mL, respectively [36]. The extract inhibited virus-induced apoptosis, inactivated viral 2A protease, and reduced the expression of IL-6 and RANTES. The extract also reduced the virus yield in the intestine of EV71-infected suckling mice. The ethyl acetate (EA) fraction of the extract showed greater antiviral activity than the n-butanol or aqueous fractions, exhibiting EC_50_ values of 4.21 µg/mL against EV71 and 9.08 µg/mL against CVA16 [37]. Eupafolin, a major component of the EA fraction, showed EC_50_ values of 1.39 µM against EV71 and 5.24 µM against CVA16. Eupafolin attenuated the virus-induced upregulation of IL-6 and RANTES by inhibiting the virus-induced ERK1/2, AP-1, and STAT3 signals.

*Saururus chinensis* (Lour.) Baill (*San Bai Cao*) exhibited a variety of bioactivities for the treatment of edema, jaundice, cancer, and inflammatory diseases [38]. The water extract of *S.chinensis* showed significant antiviral activity against EV71 with an EC_50_ of 8.9 µg/mL by inhibiting the activation of the MEK1/ERK signaling pathway, and rutin was identified as the major component responsible for this activity [36].

The bark, leaves, and flowers of *Paulownia tomentosa* (*Pao Tong*) have been applied to the treatment of infections and inflammatory diseases in CM [39]. The methanol extract of *P. tomentosa* flowers demonstrated anti-EV71 activity with a dose-dependent reduction of the CPE and an EC_50_ of 65 µg/mL. Further bioactivity-guided isolation led to the discovery of the pure anti-EV71 compound apigenin, which inhibited viral replication [40].

The EA and butanol extract of *Phyllanthus urinaria* (*Zhen Zhu Cao*) was reported to possess antiviral activity against EV71 and CVA16 based on CPE reduction assays, with corilagin identified as the major active component [41].

### Other medicines

*Salvia miltiorrhiza* (*Dan Shen*) has been widely used in CM to improve blood circulation, relieve blood stasis, and treat coronary heart disease [42]. Wu et al. [43] obtained seven extracts of *S. miltiorrhiza*, and two fractions derived from water extracts showed anti-EV71 activity in CPE inhibition assays with EC_50_ values of 0.742 mg/mL for fraction SA1 and 0.585 mg/mL for SA2. Both fractions exhibited antiviral activity against three strains of EV71 in Vero, RD, and MRC-5 cell lines. The extracts also inhibited EV71-induced plaque formation and apoptosis.

*Pueraria lobata* (*Ge Gen*) is commonly used for the treatment of cold, fever, and dysentery [44]. *P. lobata* exhibited antidiabetic, anti-inflammatory, antioxidant, and antiviral activities against respiratory syncytial virus (RSV) [45–47]. The water extract of *P. lobata*, which is the main component of *Ge Gen Tang*, inhibited the CPE induced by EV71 when given before, simultaneously with, or after infection, with an EC_50_ of 0.028 µg/mL and an SI of >107,000 in a human foreskin fibroblast cell line [48]. The extract inhibited viral attachment and penetration of the host cell and decreased EV71-induced IFN production.

*Glycyrrhiza uralensis* (*Gan Cao*) is a tonic herb with a wide range of bioactivities, such as antiulcer, anti-inflammatory, spasmolytic, antioxidative, antiviral, anticancer, and hepatoprotective effects [49]. The water extract of *G. uralensis* inhibited the EV71-induced CPE in a human foreskin fibroblast cell line with an EC_50_ of 0.056 µg/mL, and treatment after viral infection provided a better protection rate than treatment before infection [50]. The protective mechanism might have involved the prevention of viral attachment and penetration, but did not involve activation of the IFN pathway.

*Ampelopsis brevipedunculata* Trautv (*Shan Pu Tao*) is used for the treatment of liver disease and inflammation, and as a food ingredient [51]. The herb also exhibited antioxidative, hepatoprotective, and antiviral activities toward HBV [52–54]. The extracts of *A. brevipedunculata* Trautv showed the most potent anti-EV71 activity among 58 Taiwanese folk medicinal plants examined [51]. The EC_50_ of the acetone extract was 26.11 µg/mL, and the inhibitory effects may be related to effects on viral infection, activity, and replication. The extract may also significantly upregulate IL-6 and IL-1β levels and downregulate IL-8 levels.

*Daphne Genkwa* Sieb. et Zucc. (*Yuan Hua*) is used for its diuretic, anti-inflammatory, and detoxifying effects with antioxidative activity [55]. Components isolated from *D. Genkwa* exhibited anti-inflammatory and anticancer activities [56–58]. The extract of dried buds from *D. Genkwa* Sieb. et Zucc. reduced the EV71-induced CPE with EC_50_ values of 0.163–0.824 mg/mL, and exhibited strong anti-EV71 activity during the viral pre-adsorption step [59].

## Chinese medicinal formulae

*Sheng*-*Ma*-*Ge*-*Gen*-*Tang* (*SMGGT*) is a Chinese formula, consisting of four herbal medicines: *Rhizoma Cimicifugae* (*Sheng Ma*), *P. lobata* (*Ge Gen*), *Glycyrrhiza uralensis* (*Gan Cao*), and *Raeonia lactiflora* (*Shao Yao*), and it is frequently used for treatment of measles, fevers and headaches; and it exhibited antiviral effects on human respiratory syncytial virus [60]. An extract of *SMGGT* significantly inhibited EV71-induced CPE with an EC_50_ of approximately 0.21 μg/mL and without toxicity at concentrations up to 5000 μg/mL (SI > 23809.52) [61]. The extract inhibited viral attachment and penetration, though it did not significantly change the IFN level.

*GuiQi* polysaccharides (GQP) are derived from the water extract and alcohol precipitation of mixtures of *Angelica sinensis* (*Dang Gui*) and *Astragalus membranaceus* (*Huang Qi*) roots in a ratio of 1:5, and this formula is used for the tonification of *qi* and *xue* in the body. GQP significantly reduced EV71 induced-CPE with EC_50_ below 31.2 μg/mL and blocked EV71 adsorption rather than inhibiting EV71 replication [62].

## Pure compounds derived from CHMs with anti-EV71 activity

Extracts of CHMs show a variety of bioactivities and contain diverse mixtures of essential bioactive compounds. Pure compounds with anti-EV71 activity are summarized in Table 2.

**Table 2:** Anti-EV71 effects of pure compounds from Chinese medicines

Category	Compound and reference	Chemical structure	Plant source	Chemical class	Anti-EV71 effect	Mechanism
Flavones	Apigenin [65]		*Ocimum basilicum* (*Luo Le*), etc	Flavone	Reduces CPE (EC_50_ 25.5 μM, SI 8.7), viral protein expression, ROS generation, cytokine up-regulation	Interferes with viral IRES activity, JNK activation, association of EV71 RNA with hnRNP A1 and A2 proteins
	Chrysosplenetin [67]		*Laggera pterodonta* (*Chou Ling Dan*)	Flavonol	Reduces CPE (EC_50_, 0.17 μM, SI 107.5), plaque formation, production of viral VP1 protein, and the viral yield	Shows strong antiviral potency targeting the post-attachment stage
	Penduletin [67]		*Laggera pterodonta* (*Chou Ling Dan*)	Flavonol	Reduces CPE (EC_50_ 0.17 μM, SI 655.5), plaque formation, production of viral VP1 protein, and the viral yield	Shows strong antiviral potency targeting the post-attachment stage
	7,8-dihydroxyflavone [76]		*Chrysanthemum morifolium* Ramat (*Ju Hua*), etc	Flavone	Shows 20 % cytotoxicity, 80 % CPE reduction and 40 % IRES activity at 50 μM	
	Kaempferol [76]		*Chrysanthemum morifolium* Ramat (*Ju Hua*), etc	Flavonol	Shows 20 % cytotoxicity, 80 % CPE reduction and 40 % IRES activity at 50 μM, reduces virus yield, and viral protein expression	Changes the expression level of FUBP1, FUBP3, HNRPD, HNRH1 and HNRPF proteins, which may contribute to the anti-EV71 activity
	Quercetin [76]		*Chrysanthemum morifolium* Ramat (*Ju Hua*), etc	Flavonol	Shows 20 % cytotoxicity, 80 % CPE reduction and 40 % IRES activity at 50 μM	
	Hesperetin [76]		*Chrysanthemum morifolium* Ramat (*Ju Hua*), etc	Flavonone	Shows 20 % cytotoxicity, 80 % CPE reduction and 40 % IRES activity at 50 μM	
	Hesperidin [76]		*Chrysanthemum morifolium* Ramat (*Ju Hua*), etc	Flavonone glucoside	Shows 20 % cytotoxicity, 80 % CPE reduction at 50 μM	
	Eupafolin [37]		*Kalanchoe gracilis* (*Deng Long Cao*)	Flavone	Reduces CPE (EC_50_ 0.44 μM, SI 808), plaque formation, decreases virus-induced IL-6 and RANTES expression, and decreases the phosphorylation of cytokine induction-related proteins	Inactivates the virus, and suppresses proinflammatory cytokines
	Chrysin [79]		*Oroxylum indicum* (L.)Vent. (*Mu Hu Die*), *Pinus mon*-*ticola* Dougl. (*Bai Shan Song*)	Flavone	Reduces CPE (EC_50_ 10 μM, SI 20), viral RNA, capsid protein, and infectious virions	Inhibits viral 3C protease
	Chrysin phosphate ester [79]		Synthesised	Flavone derivative	Reduces CPE (EC_50_ 6 μM, SI 33), viral RNA, capsid protein, and infectious virion	Inhibits viral 3C protease
	Luteolin [64, 80]		*Lonicera japonica* (*Jin Yin Hua*), *Dendranthema indicum* (*Ye Ju Hua*)	Flavone	Reduces CPE (EC_50_ 31.56 μM, SI 9.25 in RD cells), inhibits viral RNA replication	Targets post-attachment stage
	Rutin [38]		*Saururus chinensis* (Lour.) Baill (*San Bai Cao*)	Flavonoid glycoside	Reduces CPE (200 μM), viral RNA level, and virus titre	Inhibits activation of MEK1-ERK signalling pathway
	Formononetin [82]		*Trifolium pratense (San Ye Cao)*, etc	Isoflavone	Reduces CPE (EC_50_ 3.98 μM, SI 43.07), viral RNA replication, protein synthesis	Suppresses ERK, p38, and JNK activation, and COX-2/PGE_2_ expression
Terpenes	Ursolic acid [63]		*Ocimum basilicum* (*Luo Le*)	Triterpenoid	Reduces CPE (EC_50_ 1.1 μM, SI 200)	Inhibits viral infection and replication process
	Linalool [65]		*Ocimum basilicum* (*Luo Le*)	Monoterpene	Reduces CPE (EC_50_ 273.60 μM, SI 4.2)	
	Raoulic acid [84]		*Raoulia australis*	Diterpene	Reduces CPE (EC_50_ 0.25 μM, SI above 658)	
	Glycyrrhizic acid [87]		*Glycyrrhiza uralensis* (*Gan Cao*)	Triterpenoid	Reduces plaque formation at 3, 5 μM and virus titre and expression of viral VP1 protein	Targets post-viral entry process
	Geniposide [88]		*Fructus gardeniae* (*Zhi Zi*)	Monoterpene	Reduces CPE, viral RNA level, plaque formation, and inhibited viral IRES activity	
	GLTA [91]		*Ganoderma lucidum* (*Ling Zhi*)	Triterpenoid	Reduces CPE (EC_50_ below 0.16 μg/mL)	Blocks adsorption and uncoating
	GLTB [91]		*Ganoderma lucidum* (*Ling Zhi*)	Triterpenoid	Reduces CPE (EC_50_ below 0.16 μg/mL)	Blocks adsorption and uncoating
	Hederasaponin B [92]		*Hedera helix* (*Chang Chun Teng*)	Triterpenoid	Reduces CPE (EC_50_ 24.77 μM, SI 2.02) and viral capsid protein expression	Inhibits viral capsid protein expression
	Ginsenoside Rg2 [95]		*Panax ginseng* Meyer (*Ren Shen*)	Triterpenoid	Reduces CPE	
Polyphenols	Epigallocatechin gallate (EGCG) [96]		*Camellia sinensis* (*Lv Cha*)	Polyphenol	Reduces plaque formation, viral RNA level, and raises the survival rate of Vero cells approximately fourfold relative to untreated infected cells at 25 μM	Has antioxidant activity, and suppresses viral RNA replication
	Gallocatechin gallate (GCG) [96]		*Camellia sinensis* (*Lv Cha*)	Polyphenol	Reduces plaque formation, and raises the survival rate approximately fourfold higher than the infected group at 25 μM	
	Geraniin [99]		*Geranium thunbergii* (*Lao Guan Cao*)	Tannin	Reduces CPE, viral yield, can improve survival and clinical score in infected mice (EC_50_ 10.5 μM, SI 20)	
	Chebulagic acid [102]		*Terminalia chebula* (*He Zi*)	Tannin	Reduces CPE, and reduces the mortality of infected mice, relieves the symptoms (EC_50_ 13.1 μM, SI 16)	Inhibits viral replication
	Corilagin [41]		*Phyllanthus urinaria* (*Zhen Zhu Cao*)	Ellagitannins	Reduces CPE (EC_50_ 5.6 μg/mL)	
	Punicalagin [103]		*Punica granatum* L. (*Shi Liu*)	Tannin	Reduces CPE (EC_50_ 15 μg/mL), viral RNA level, and mice mortality in vivo	
Steroids	Timosaponin B-II [105]		*Anemarrhena asphodeloides* (*Zhi Mu*)	Steroidal saponin	Reduces CPE (EC_50_ 4.3 μM, SI 92.9)	
	Anemarrhenasaponin II [105]		*Anemarrhena asphodeloides* (*Zhi Mu*)	Steroidal saponin	Reduces CPE (EC_50_ 22.2 μM, SI 3.8)	
	Timosaponin G [105]		*Anemarrhena asphodeloides* (*Zhi Mu*)	Steroidal saponin	Reduces CPE (EC_50_ 9.1 μM, SI 2.3)	
	Timosaponin A-IV [105]		*Anemarrhena asphodeloides* (*Zhi Mu*)	Steroidal saponin	Reduces CPE (EC_50_ 4.7 μM, SI 2.2)	
	Timosaponin A-III [105]		*Anemarrhena asphodeloides* (*Zhi Mu*)	Steroidal saponin	Reduces CPE (EC_50_ 1.1 μM, SI 2.4)	
	Shatavarin IV [105]		*Anemarrhena asphodeloides* (*Zhi Mu*)	Steroidal saponin	Reduces CPE (EC_50_ 2.2 μM, SI 1.8)	
Miscellaneous	Gallic acid [113]		*Woodfordia fruticosa* (*Xia Zi Hua*)	Phenolic acid	Reduces CPE (EC_50_ 4.47 μM, SI 99.57)	Has antioxidant activity
	Resveratrol [116, 117]		*Vitis vinifera* L. (*Pu Tao*), *Polygonum cuspidatum* Sieb.et Zucc.(*Hu Zhang*), *Fructus mori* (*Sang Shen*), *Arachis hypogaea* Linn. (Hua Sheng), *Veratrum grandiflorum* (*Mao Ye Li Lu*)	Phenol	Reduces CPE (EC_50_ 20.2 mM, SI 15.2)	Blocks IKKs/NF-κB signalling pathway
	Allophycocyanin [120]		*Spirulina* *platensis*	Protein	Reduces CPE (EC_50_ 0.045 μM, SI 36.7), plaque formation (EC_50_ 0.056 μM, SI 29.5), delays viral RNA synthesis, and inhibits EV71-induced apoptosis	Interferes with early stage of viral replication
	Caffeic acid [36]		*Kalanchoe gracilis* (*Deng Long Cao*)	Phenol	Reduces CPE (EC_50_ 23.87 μM, SI 11.51), plaque formation	
	Aloe-emodin [124]		*Rheum palmatum* (*Da Huang*)	Anthraquinone	Induces IFN expression, activates NO production, and reduces plaque formation (EC_50_ 0.5-1.9 μM, SI above 5540)	Activates type I and II IFN signalling pathways against viral replication
	Garlicin [127]		*Allium Sativum* (*Da Suan*)	Diallyl disulfide	Reduces CPE (EC_50_ 99.95 μM, SI 44.66)	
	Oblongifolin J [128]		*Garcinia oblongifolia* (*Ling Nan Shan Zhu Zi*)	Prenylated benzoylphloroglucinol	Reduces CPE (EC_50_ 31.1 μM, SI 1.5)	
	Oblongifolin M [128]		*Garcinia oblongifolia* (*Ling Nan Shan Zhu Zi*)	Prenylated benzoylphloroglucinol	Reduces CPE (EC_50_ 16.1 μM, SI 2.4)	
	Euxanthone [128]		*Garcinia oblongifolia* (*Ling Nan Shan Zhu Zi*)	Xanthone	Reduces CPE (EC_50_ 12.2 μM, SI 3.0)	
	Gramine derivative 4 s [130]		Synthesised	Indole alkaloid	Reduces CPE (EC_50_ 9.1 μM, SI 14.3), viral RNA replication, protein synthesis, and virus-induced apoptosis	Inhibits viral adsorption or affects viral release from the cells
	Chlorogenic acid [131]		*Lonicera japonica* (*Jin Yin Hua*), *Eucommia ulmoides* Oliv. (*Du Zhong*), *Lythrum salicaria L.* (*Qian Qu Cai*)	Aromatic acids	Reduces plaque formation (EC_50_ 6.3 μg/mL)	Inhibits EV71 2A transcription and translation
	Magnesium lithospermate B [132]		*Salvia miltiorrhiza* (*Dan Shen*)	Aromatic acids	Reduces CPE (EC_50_ 0.09 mM, SI 10.52), plaque formation, protein expression	Influences virus infection, and IRES activity
	Rosmarinic acid [132]		*Salvia miltiorrhiza* (*Dan Shen*)	Aromatic acids	Reduces CPE (EC_50_ 0.50 mM, SI 2.97), plaque formation, protein expression	Influences virus infection, and IRES activity
	Matrine [137]		*Sophora flavescens* (*Ku Shen*)	Gordon landmines ketoneses alkaloid	Reduces viral RNA level, and mice mortality in vivo	
	Lycorine [145]		*Lycoris radiata* (*Shi Suan*)	Benzylphenethylamine alkaloid	Reduces CPE (EC_50_ 0.48 μg/mL, SI above 100), viral RNA level, and mice mortality in vivo	Influences viral protein expression

### Flavones

Apigenin is widely distributed in a variety of plants, such as *Ocimum basilicum* (*Luo Le*), parsley, artichoke, basil, and celery. Apigenin showed anti-EV71 activity at approximately 25 µM, and inhibited viral protein expression, reactive oxygen species (ROS) generation, and cytokine upregulation [63, 64]. Apigenin also interfered with viral internal ribosome entry site (IRES) activity and JNK activation [65]. The study also suggested that apigenin inhibited the association of EV71 RNA with RNA-editing-related hnRNP proteins [65].

Chrysosplenetin and penduletin are two flavonols isolated from the leaves of *Laggera pterodonta* (*Chou Ling Dan*), which is used for *clearing heat* and *detoxification* [66]. These flavonols exhibited potent anti-EV71 activity with low EC_50_ values of 0.20 µM for chrysosplenetin and 0.17 µM for penduletin, and had SI values of >100 [67]. The flavonols showed strong antiviral potency by targeting the viral post-attachment stage. Flavonoids with 3-methoxy, 5-hydroxy, and 4′-hydroxyl groups showed antipicornavirus activity by targeting the phosphatidylinositol 4-kinase IIIβ (PI4KB) pathway, and being within the same group, chrysosplenetin and penduletin might inhibit PI4KB, which would contribute to their anti-EV71 activity [68]. The PI4KB/oxysterol-binding protein (OSBP) pathway was the major target of the anti-picornavirus activity of enviroxime-like compounds and flavonoids with 3-methoxy, 5-hydroxy, and 4′-hydroxyl groups [69–72]. Inhibition of the PI4 KB/OSBP pathway might contribute to the anti-EV71 activity of many uncharacterized compounds [69, 73, 74]. However, as reported, consecutive administration of two different structural PI4 KB inhibitors in SJL mice exhibited certain toxicity, which limited their further application [75].

7,8-dihydroxyflavone, kaempferol, quercetin, hesperetin, and hesperidin are polyphenolic flavones with inhibitory effects on EV71 infection at a concentration of 50 µM [76]. Among them, 7,8-dihydroxyflavone, kaempferol, and hesperetin inhibited 40 % of viral IRES activity. Kaempferol also significantly reduced the viral yield by its regulatory effects on IRES function and EV71 replication through changes to the IRES-associated trans-acting factors FUBP1, FUBP3, HNRPD, HNRH1, and HNRPF.

Chrysin (CR) is a flavone extracted from the seeds of *Oroxylum indicum* (L.) Vent. (*Mu Hu Die*) and other plants, and exhibited antitumor and antidiabetic bioactivities [77, 78]. CR was indicated to show possible binding to EV71 protease 3C in Autodock 4.0 simulations [79]. CR exhibited strong anti-EV71 activity with an EC_50_ of 10 µM in CPE inhibition assays, while its phosphate ester (CPI) showed a more potent effect with a lower EC_50_ of 6 µM.

Luteolin can be found in many plants, such as *Lonicera japonica* (*Jin Yin Hua*) and *Perilla frutescens* (L.) Britt (*Bai Su*). This flavonoid exhibited various pharmacological activities, including inhibition of EV71 and CVA16 with EC_50_ values of approximately 10 µM [80]. Xu et al. [80] reported that luteolin targeted the post-attachment stage of EV71 and CVA16 infection by inhibiting viral RNA replication, while Lv et al. [64] reported that it might act on viral polyprotein expression after viral entry of EV71, and prevent EV71-induced cell apoptosis, intracellular ROS generation, and cytokine upregulation.

Formononetin can be extracted from many herbs and plants, such as leguminous plants [81]. It exhibited various bioactivities including anti-inflammatory, antioxidative, and anticancer effects [82]. In a large-scale screening, formononetin demonstrated significant anti-EV71 activity [82]. Specifically, it inhibited the EV71-induced CPE with an EC_50_ of 3.98 µM, reduced virus RNA replication and protein expression in a dose-dependent manner, and exerted antiviral activity by application before and after EV71 infection. The mechanism of the formononetin activity involved the suppression of ERK, p38 MAPK, and JNK activation as well as the suppression of EV71-induced COX-2/PGE_2_ expression.

### Terpenes

Raoulic acid is the main component of *Raoulia australis*, a perennial shrub plant from New Zealand. Raoulic acid reduced the EV71-induced CPE [83], and possessed broad-spectrum antiviral activity against six HRVs with EC_50_ values of less than 0.1 µg/mL [84].

Ursolic acid from *O. basilicum* was reported to possess antitumor activity [85]. It showed strong anti-EV71 activity with an EC_50_ of 1.1 µM and SI of >200, and might inhibit viral infection and replication processes [63].

Glycyrrhizic acid is a major bioactive compound found in *G. uralensis* (*Gan Cao*), which is used for the treatment of sore throat, cough, peptic ulcers, and other ailments in CM [49]. The compound exhibited anti-inflammatory, antidiabetic, antioxidative, anticancer, anti-microbial, and antiviral properties [86]. Glycyrrhizic acid suppressed the EV71-induced CPE and plaque formation at 3 and 5 mM, respectively, and might target post-viral entry processes [87].

Geniposide is a primary component of *Fructus gardeniae* (*Zhi Zi*), a fruit that can be used for its laxity and anti-inflammatory effects [88]. Geniposide protected more than 80 % of cells against EV71 infection at a concentration of 3 mg/mL, and reduced the EV71-induced CPE by approximately 80 % at a concentration of 2 mg/mL [89]. Geniposide might block the translation of viral proteins.

Lanosta-7,9(11),24-trien-3-one,15,26-dihydroxy (GLTA) and ganoderic acid Y (GLTB) are two triterpenoids from *Ganoderma lucidum* (*Ling Zhi*), which is widely used in CM to treat a variety of diseases and has potential for bioremediation [90]. These triterpenoids reduced the EV71-induced CPE with EC_50_ values of <0.16 µg/mL and blocked viral particle uncoating [91].

Hederasaponin B can be isolated from *Hedera helix* (*Chang Chun Teng*). The isolate inhibited the CPE induced by the C3 and C4a types of EV71 with EC_50_ values of <0.16 µg/mL and reduced viral capsid protein expression [92].

Ginsenosides are major active components of *Panax ginseng* Meyer (*Ren Shen*). Ginsenosides possesses anti-aging, antidiabetic, anticancer, and antiviral activities [93, 94]. In a cell-based screening of seven ginsenosides, a CPE reduction assay was applied and quantified with the sulforhodamine B method [95]. Among the substances examined in the screening, only ginsenoside Rg2 showed moderate dose-dependent anti-EV71 effects.

### Polyphenols

Epigallocatechin gallate (EGCG) and gallocatechin gallate (GCG) are two tea catechins that significantly reduced EV71-induced plaque formation, while EGCG also reduced the viral RNA levels of EV71 [96]. EGCG and GCG at concentrations of 25 µM increased the cell survival rate by approximately fourfold compared with the rate in mock-infected Vero cells. The anti-oxidative activity of EGCG might contribute to the anti-EV71 activity.

Geraniin derived from *Geranium thunbergii* (*Lao Guan Cao*) possessed anti-bacterial, anti-diarrheal, antioxidative, and anti-hypertensive effects, and induced cell death [97, 98]. Geraniin reduced the EV71-induced CPE in vitro with an EC_50_ of 10.5 µM and improved the survival rate and clinical score of EV71-infected mice [99].

Chebulagic acid, a hydrolysable tannin, is isolated from the fruits of *Terminalia chebula* (*He Zi*), and used for its spasmolytic, anti-diarrheal, anti-bacterial, anti-hyperglycemic, and broad-spectrum antiviral activities [100, 101]. Chebulagic acid showed anti-EV71 activity in vitro with an EC_50_ of 13.1 µM, and reduced the mortality and relieved the symptoms of EV71-infected mice by inhibiting viral replication in vivo [102].

Punicalagin was examined for its antiviral effects, and reduced both the CPE and viral RNA levels in vitro with an EC_50_ of 15 µg/mL. Furthermore, punicalagin reduced the mortality and relieved the clinical symptoms, such as hind limb paralysis, of mice in vivo [103].

### Steroids

Components of *Anemarrhena asphodeloides* (*Zhi Mu*) exhibited significant pharmacological effects on the nervous system and blood, and displayed antitumor, antioxidative, antimicrobial, antiviral, anti-inflammatory, antiosteoporotic, skin-protective, and anti-aging effects [104]. By applying an isolation method called folding fan mode counter-current chromatography and CPE reduction assays, six anti-EV71 saponins were identified in *A. asphodeloides*, among which timosaponin B-II displayed the best medicinal potential with an EC_50_ of 4.3 µM and the highest SI of 92.9 [105]. Further improvements of the isolation method were achieved using two-phase solvent systems in sample pre-treatment, which increased the production yield of the active compound [106].

### Miscellaneous

Gallic acid is a component of *Woodfordia fruticosa* flowers (*Xia Zi Hua*), which are used to treat dysentery and irregular menstruation, and exhibited antibacterial, hepatoprotective, and immunostimulatory effects [107–109]. Gallic acid also exhibited antibacterial, anti-inflammatory, antiallergic, and neuroprotective effects [110–112] and showed an inhibitory effect on the EV71-induced CPE in Vero cells with an EC_50_ of 4.47 µM [113].

Resveratrol is contained in grapes, mulberries, peanuts, *Polygonum cuspidatum* (*Hu Zhang*), and several other sources. Resveratrol exhibited antioxidant and anti-inflammatory activities and improved glucose and lipid metabolism [114]. Resveratrol also acted on cardiovascular parameters and modified some pathways involved in carcinogenesis [115]. Resveratrol increased the survival rate of EV71-infected Vero cells with an EC_50_ of 20.2 mM and SI of 15.2 [116]. Resveratrol inhibited the virus titer and protein expression by blocking the IKK/NF-κB signaling pathway [117]. However, polydatin is the most abundant form of resveratrol that exists naturally, and this compound did not show significant anti-EV71 activity.

Allophycocyanin is a fluorescent protein derived from the blue-green alga *Spirulinaplatensis*, and exhibited antioxidant and anticancer activities [118, 119]. The protein exerted anti-EV71 activity in CPE and plaque reduction assays with an EC_50_ of 0.045 µM, delayed viral RNA synthesis, and inhibited EV71-induced apoptosis [120]. Allophycocyanin also showed antiviral activity against CVA16.

Aloe-emodin is a free anthraquinone isolated from *Rheum palmatum* (*Da Huang*) that is used for purgation, *clearing heat*, and *detoxification*, and exhibited antibacterial, hepatoprotective, antitumor and antiangiogenic effects [121–123]. Aloe-emodin showed antiviral activity against EV71 in HL-CZ and TE-671 cells with EC_50_ values of 0.5–1.9 µM [124]. Aloe-emodin induced the expression of IFNs, and might be involved in the activation of the type I and II IFN signaling pathways against viral replication.

Garlicin, a component of *Allium sativum* (*Da Suan*), is a commonly used food ingredient in Asia. *A. sativum* has antimicrobial, anticancer, antidiabetic, anti-fatigue, and blood pressure-reducing effects [125, 126]. Garlicin inhibited the EV71-induced CPE in Vero cells with an EC_50_ of 99.95 µM [127].

Oblongifolin J, oblongifolin M, and euxanthone are isolated from the leaves of *Garcinia oblongifolia* Champ. ex Benth (*Ling Nan Shan Zhu Zi*), which have anti-inflammatory and analgesic activities and can be used to treat myogenic convergence, allergies, rash, itching, ulcers, hepatitis, laryngitis, and hemoptysis. The isolates inhibited the CPE in EV71-infected Vero cells with EC_50_ values of 31.1, 16.1, and 12.2 µM, respectively [128].

Gramine, a natural indole alkaloid, can be isolated from various raw plants and coal tar, and exhibited broad pharmaceutical activities, such as relaxation of bronchial smooth muscle, vasorelaxation, blood pressure elevation, relief of bronchitis nephritis, and relief of bronchial asthma [129]. Gramine did not exhibit anti-EV71 activity [129]. However, Wei et al. [130] performed a series of chemical modifications on gramine, and showed that 18 of 21 derivatives displayed some degree of anti-EV71 effect. Among the derivatives, 4 s had a relatively low EC_50_ of 9.1 µM and the highest SI of 14.3. In their study, 4 s inhibited the virus-induced cell apoptosis, viral RNA replication, and viral protein expression, and may therefore target the early stage of the EV71 lifecycle.

Chlorogenic acid is a major active component of many CHMs, including *Eucommia ulmoides* Oliv. (*Du Zhong*), *L. japonica* Thumb. (*Jin Yin Hua*), and *Polygonum aviculare* L. (*Bian Xu*). Chlorogenic acid reduced EV71-induced plaque formation with an EC_50_ of 6.3 µg/mL, inhibited viral protein 2A transcription and translation, and downregulated IL-6, TNF-α, IFN-γ, and MCP-1 secretion in EV71-infected RD cells [131].

Magnesium lithospermate B (MLB) and rosmarinic acid (RA) are two compounds found in *S. miltiorrhiza*. The results from pGS-EV71 IRES-based bicistronic reporter assays suggested that MLB and RA inhibited EV71 IRES activity [132], and further inhibited the EV71-induced CPE with EC_50_ values of 0.09 and 0.50 mM, plaque formation, and viral protein expression. These compounds exerted their antiviral effects during the viral absorption stage.

Matrine is isolated from *Sophora flavescens* (*Ku Shen*), and used for its *heat*-*clearing* and *detoxifying* properties. Matrine exhibited anticancer, antidiabetic, hepatoprotective, and cardioprotective effects [133–136]. It also showed significant inhibitory effects on EV71 in vitro and in vivo [137]. Matrine reduced the viral RNA levels in RD cells, and protected mice from a lethal dose of EV71 virus while relieving the clinical symptoms of infection.

As one of the most abundant alkaloids in the* Amaryllidaceae* family, lycorine exhibited anticancer and anti-inflammatory properties and conferred antiviral effects against human immunodeficiency virus (HIV), hepatitis C virus, and HSV-1 [138–144]. In RD cells, lycorine exhibited a dose-dependent reduction of the EV71-induced CPE with an EC_50_ of 0.48 µg/mL. Treatment of cells with 1.0 µg/mL lycorine significantly inhibited the viral RNA level. The antiviral mechanism might be related to interference with viral polyprotein translation. Lycorine inhibited EV71 replication in muscle tissues of mice, resulting in reduced mortality, dose-dependent increases in clinical scores, and reduced pathological changes including virions in tissues, moderate inflammation, and necrotizing myositis in muscle [145].

## Application of CM in treatment of HFMD

In the past three decades, there have been several outbreaks of HFMD in Taiwan, Singapore, Australia, Japan, and China, leading to millions of infections and thousands of deaths [2, 146–149]. EV71 is mainly responsible for the severe symptoms caused by HFMD. No specific antiviral agent is available, making clinical management of HFMD largely supportive in nature [150]. The Ministry of Health of China issued “Guidelines for the diagnosis and treatment of hand, foot, and mouth disease” in 2010, and recommended a series of CHMs for the treatment of HFMD [151].

According to CM syndrome differentiation, HFMD has been classified into different groups with corresponding CM treatment recommendations (Table 3).

**Table 3:** Chinese medicines recommended for the treatment of HMFD by the Chinese government

Classification of HMFD	CM syndrome	Chinese medicine	Composition
General	*Dampness* and *heat* in *lung* and *spleen*	*Gan Lu Xiao Du Dan* decoction	*Forsythia suspense* (*Lian Qiao*), *Lonicera japonica* (*Jin Yin Hua*), *Scutellaria baicalensis* (*Huang Qin*), *Artemisia apiacea* (*Qing Hao*), *Fructus Arctii* (*Niu Bang Zi*), *Agastache rugosa* (*Huo Xiang*), *Eupatorium fortune* (*Pei Lan*), *Ricepaperplant Pith* (*Tong Cao*), barley (*Yi Mi*), talcum (*Hua Shi*), *Glycyrrhiza uralensis* (*Gan Cao*), *Imperata cylindrical* (*Bai Mao Gen*)
		*Lan Qin* oral liquid	*Indigowoad* root (*Ban Lan Gen*), *Scutellaria baicalensis* (*Huang Qin*)*, Gardenia jasminoides* Ellis (*Zhi Zi*)*, Phellodendron amurense* (*Huang Bai*)*, Sterculia lychnophora* (*Pang Da Hai*)
		*Xiao Er Chi Qiao Qing Re* granule	Forsythia suspense (*Lian Qiao*), *Semen Sojae Praeparatum* (*Dan Dou Chi*), *Mentha haplocalyx* (*Bo He*), *Schizonepeta* (*Jing Jie*), *Gardenia jasminoides* Ellis (*Zhi Zi*), *Rheum rhabarbarum* (*Da Huang*), *Artemisia apiacea* (*Qing Hao*), red peony root (*Chi Shao*), *Areca catechu* (*Bing Lang*), *Mangnolia officinalis*(*Hou Po*), *Scutellaria baicalensis* (*Huang Qin*), *Pinellia ternate* (*Ban Xia*), *Bupleurum chinense* (*Chai Hu*), *Glycyrrhiza uralensis* (*Gan Cao*)
		*Jin Lian Qing Re* effervescent tablets	*Trollius chinensis* (*Jin Lian Hua*), *Folium isatidis* (*Da Qing Ye*), gypsum (*Shi Gao*), *Anemarrhena asphodeloides* (*Zhi Mu*), *Scrophularia ningpoensis* (*Xuan Shen*), *Semen armeniacae amarae* (*Ku Xing Ren*)
		*Kang Bing Du* oral liquid	Indigowoad root (*Ban Lan Gen*), gypsum (*Shi Gao*), *Arbados aloe* (*Lu Hui*), *Rehmannia glutinosa* (*Sheng Di Huang*), *Curcuma aromatic* (*Yu Jin*), *Anemarrhena asphodeloides* (*Zhi Mu*), *Acorus gramineus* (*Shi Chang Pu*), *Pogostemon cablin* (*Guang Huo Xiang*), *Forsythia suspense* (*Lian Qiao*)
	Stagnation and steaming of *damp*-*heat*	*Qing Wen Bai Du* decoction	*Forsythia suspense* (*Lian Qiao*), *Gardenia jasminoides* Ellis (*Zhi Zi*), *Scutellaria baicalensis* (*Huang Qin*), *Coptis chinensis* (*Huang Lian*), gypsum (*Shi Gao*), *Anemarrhena asphodeloides* (*Zhi Mu*), *Salivia chinensis* (*Dan Pi*), red peony root (*Chi Shao*), barley (*Yi Mi*), *Dioscoreae hypoglaucae* (*Chuan Bi Xie*), buffalo horn (*Shui Niu Jiao*)
		*Xin Xue Dan*	Lodestone (*Ci Shi*), gypsum (*Shi Gao*), talcum (*Hua Shi*), gypsum rubrum (*Han Shui Shi*), saltpeter (*Xiao Shi*), mirabilite (*Mang Xiao*), *Gardenia jasminoides* Ellis (*Zhi Zi*), *Lophatherum gracile* (*Dan Zhu Ye*), *Cimicifugae foetidae* (*Sheng Ma*), *Andrographis paniculata* (*Chuan Xin Lian*), pearl powder(*Zhen Zhu Ceng Fen*), *Lignum aquilariae resinatrm* (*Chen Xiang*), calculus bovis (*Niu Huang*), borneol (*Bing Pian*)
		*Re Du Ning* injection	*Artemisia apiacea* (*Qing Hao*), *Lonicera japonica* (*Jin Yin Hua*), *Gardenia jasminoides* Ellis (*Zhi Zi*)
		*Xi Yan Ping* injection	Andrographolide sulfonate
		*Dan Shen* injection	*Salvia miltiorrhiza* (*Dan Shen*)
Severe	*Toxic*-*heat* stirring *wind*	*Ling Yang Gou Teng* decoction	*Cornu saigae tataricae* (*Ling Yang Jiao*), *Uncaria tomentosa* (*Gou Teng*), *Gastrodia elata* (*Tian Ma*), gypsum (*Shi Gao*), *Coptis chinensis* (*Huang Lian*), *Gardenia jasminoides* Ellis (*Zhi Zi*), *Rheum rhabarbarum* (*Da Huang*), *Flos chrysanthemi* (*Ju Hua*), barley (*Yi Mi*), *Buthus martensi kirsch* (*Quan Xie*), silkworm larvae (*Bai Jiang Can*), concha ostreae (*Sheng Mu Li*)
		*An Gong Niu Huang Wan*	Calculus bovis (*Niu Huang*), buffalo horn (*Shui Niu Jiao)*, musk (*She Xiang*), borneol (*Bing Pian*), pearl (*Zhen Zhu*), cinnabar (*Zhu Sha*), realgar (*Xiong Huang*), *Coptis chinensis* (*Huang Lian*), *Scutellaria baicalensis* (*Huang Qin*), *Gardenia jasminoides* Ellis (*Zhi Zi*), Curcuma aromatic (*Yu Jin*)
		*Xin Xue Dan*	See above
		*Re Du Ning* injection	See above
		*Tan Re Qing* injection	*Scutellaria baicalensis* (*Huang Qin*), bear gall powder (*Xiong Dan Fen*), cornu gorais (*Shan Yang Jiao*), *Lonicera japonica* (*Jin Yin Hua*), *Forsythia suspense* (*Lian Qiao*)
		*Xi Yan Ping* injection	See above
Urgent	Fading in *heart*-*Yang*, and *lung qi*	*Shen Fu* decoction	*Panax ginseng* (*Ren Shen*), *Aconiti carmichaeli* (*Fu Zi*), *Cornus officinalis* (*Shan Zhu Yu*)
		*Shen Mai* injection	*Radix Ginseng rubra* (*Hong Shen*), *Ophiopogon japonicas* (*Mai Dong*)
		*Shen Fu* injection	*Radix Ginseng rubra* (*Hong Shen*), *Aconiti carmichaeli* (*Fu Zi*)
Recovering	Insufficient *qi yin* and residue of pathogenic factors	*Sheng Mai San*	*Panax ginseng* (*Ren Shen*), *Schisandra chinensis* (*Wu Wei Zi*), *Ophiopogon japonicas* (*Mai Dong*), *Polygonatum odoratum* (*Yu Zhu*), *Artemisia apiacea* (*Qing Hao*), *Cydonia lagenaria* Lois. (*Mu Gua*), *Radix clematidis* (*Wei Ling Xian*), *Angelica sinensis* (*Dang Gui*), loofah sponge (*Si Gua Luo*), *Glycyrrhiza uralensis* (*Gan Cao*)
Surgical	Oropharyngeal ulcer	*Qing Dai San*	Borneol (*Bing Pian*), *Mentha haplocalyx* (*Bo He*), *Acacia catechu* (*Er Cha*), *Glycyrrhiza uralensis* (*Gan Cao*), *Coptis chinensis* (*Huang Lian*), borax (*Peng Sha*), *Indigo naturalis* (*Qing Dai*), *Depositum urinae* Hominis (*Ren Zhong Bai*)
		*Shuang Liao Hou Feng San*	Pearl (*Zhen Zhu*), calculus bovis (*Niu Huang*), borneol (*Bing Pian*), *Coptis chinensis* (*Huang Lian*), *Sophorae subprostratae* (*Shan Dou Gen*), *Glycyrrhiza uralensis* (*Gan Cao*), Indigo naturalis (*Qing Dai*), *Depositum urinae* Hominis (*Ren Zhong Bai*), gypsum rubrum (*Han Shui Shi*)
		*Bing Peng San*	Borneol (*Bing Pian*), borax (*Peng Sha*), cinnabar (*Zhu Sha*), weathered sodium sulfate (*Xuan Ming Fen*)

For general cases, decoctions with effects of *clearing heat*, *dampness*, and *toxicity* were recommended, including *Forsythia suspense* (*Lian Qiao*), *L. japonica* (*Jin Yin Hua*), *Scutellaria baicalensis* (*Huang Qin*), *Artemisia apiacea* (*Qing Hao*), and *Fructus arctii* (*Niu Bang Zi*), as well as tonic medicines such as *G. uralensis* (*Gan Cao*) and red peony root (*Paeoniae rubrathe*) (*Chi Shao*), and diuretic medicines such as barley (*Semen coicis*) (*Yi Mi*). For severe cases involving symptoms such as convulsions, CM preparations that soothe the nerves were recommended, such as *Uncaria tomentosa* (*Gou Teng*), *Gastrodia elata* (*Tian Ma*), silkworm larvae (*Bai Jiang Can*), and concha ostreae (*Ostrea gigas thunberg*) (*Sheng Mu Li*). In urgent cases, potent tonic medicines such as *P. ginseng* (*Ren Shen*) and *Aconiti carmichaeli* (*Fu Zi*) were recommended for use with caution. For oropharyngeal ulcers, several CM powders were recommended to alleviate the symptoms, and for HFMD-induced combined flaccid paralysis associated with the recovery period, acupuncture and massage were recommended for inclusion in the treatment.

Although the Chinese Government recommended these CMs in the clinical treatment of HFMD, insufficient evidence is available to support their extensive application.

*Xi Yan Ping* injection is composed mainly of andrographolide sulfonate, which is used clinically to treat bronchitis, amygdalitis, and bacillary dysentery. Andrographolide is a diterpene lactone with a variety of bioactivities, including anti-inflammatory, anticancer, and immunoregulatory effects, and is isolated from *Andrographis paniculata* (Burm) Nees. (*Chuan Xin Lian*), a *heat*-*clearing* and *detoxifying* medicine. Andrographolide prevented infectious diseases by inhibiting the multiplication of infectious agents, including *Pseudomonas aeruginosa*, *Escherichia coli*, *Candida albicans*, influenza virus, RSV, and adenovirus [152]. In 2012, a review article on the clinical application and research progress of CMs in the treatment of HFMD was published [153], in which the research progress on application of CM to the treatment of HFMD was systematically analyzed. In their study, 76 trials were included, and 18 trials met the criteria for inclusion in a meta-analysis. Compared with conventional therapy, *Xi Yan Ping* injection significantly reduced the time required for fever clearance and the skin eruption-eliminating time, with no obvious side effects [154]. A further study provided evidence that andrographolide sulfonate decreased ROS production in vitro by inhibiting lipopolysaccharide-stimulated neutrophil activation, while at 5 days post-medication with andrographolide sulfonate, the plasma myeloperoxidase, S100A8/A9, histone, and IL-6 levels were markedly lower in the combination therapy group than in the conventional therapy group [155].

*Yan Hu Ning* injection is composed of potassium sodium dehydroandrographolide succinate, with defervescent, antibacterial, antiviral, and sedative effects. This injection promoted adrenal function in the treatment of upper respiratory tract infection, viral pneumonia, and child epidemic parotitis [156–158]. *Yan Hu Ning* injection reduced the time required for fever clearance and rash subsidence [153].

*Re Du Ning* injection, which is included in three *heat*-*clearing* and *detoxifying* medicines, *Qing Hao*, *Jin Yin Hua*, and *Zhi Zi*, has been studied for its antiviral and anti-inflammatory effects [159, 160]. *Re Du Ning* injection appeared to significantly reduce the time required for fever clearance and rash subsidence compared with conventional therapy [161].

*Pu Di Lan* is prepared as oral tablets or a liquid, and mainly consists of *Taraxacum mongolicum* (*Pu Gong Ying*), *S. baicalensis* (*Huang Qin*), *Corydalis bungeana* Turcz. (*Ku Di Ding*), and *Baphicacanthis cusiae Rhizoma et Radix* (*Ban Lan Gen*). *Pu Di Lan* treatment possessed superior efficacy compared with conventional drug therapy in the time required for fever clearance, rash subsidence, and oral ulcer treatment [153].

## Non-Chinese medicine

### Kappa carrageenan

Kappa carrageenan is water-soluble, sulfated galactan existing in a variety of seaweeds and is widely applied as a food additive [162]. It showed anti-EV71 activity with little associated toxicity [163]. The anti-EV71 activity might be attributable to the direct binding of carrageenan with virus particles.

### Perspectives

**Fig. 1:**
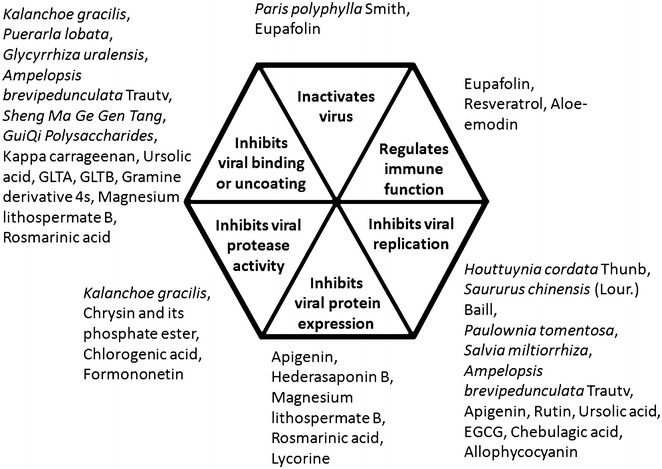
Mechanisms of anti-EV71 components. Mechanisms of anti-EV71 activities associated with the shown CM-derived extracts and molecules were classified into six categories

**Fig. 2:**
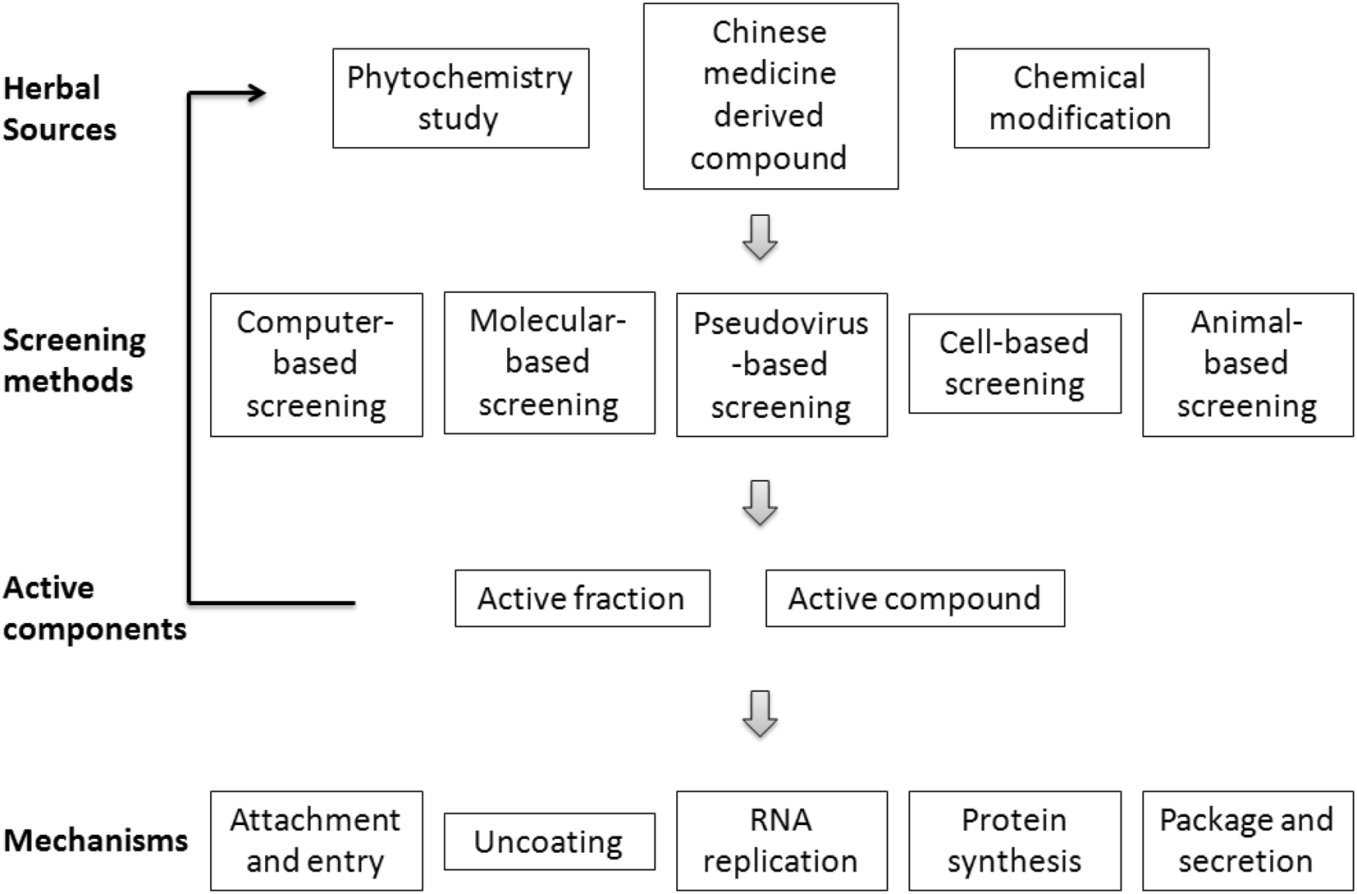
Schematic diagram of the strategies used to assess anti-EV71 activities of Chinese herbal medicines. Shown are different methods that are used to screen for anti-EV71 activity in fractions and compounds from Chinese herbal medicines. The anti-EV71 mechanisms target different points in the EV71 life cycle

In recent years, attention has been increasingly drawn to the screening of natural products and especially CM for anti-EV71 active components. The anti-EV71 mechanisms of action of these components have been summarized in Fig. 1. Common strategies for identifying anti-EV71 components are shown in Fig. 2. One strategy is bioactivity-guided isolation, which has been represented in the isolation of *Garcinia oblongifolia*, *Hedera helix* and *Anemarrhena asphodeloides*. Another strategy is systematic identification of the chemical constituents from a natural source and subsequent testing of each purified compound for antiviral activity, with more time and cost consumption. Additionally, in the case where the active compound is known and a standard substance is available, a fingerprint profile can be used for identification of them in active extracts. Furthermore, chemical modification of known active natural compounds may lead to better structural optimization to yield higher efficiency and lower toxicity, thus promoting anti-EV71 drug development [130].

Development of computer simulations allows the in-depth study of EV71 infection and pathogenic mechanisms; with the expansion of compound libraries, the anti-EV71 compound targeting of viral proteins becomes increasingly predictable, thus enabling direct synthesis of predicted bioactive molecules [79]. An alternative for cell-based screening involves a two-step platform that uses two types of reporter viruses, specifically a pseudovirus with luciferase-encoding RNA replicons encapsulated by viral capsid proteins and a full-length reporter virus expressing enhanced green fluorescent protein. The two reporters can be used to screen for possible hits and then to conduct a cell-based assay to confirm the activity [80].

Current research on anti-EV71 drug development is mostly carried out in vitro with a few in vivo studies. The current evidence for the clinical application of CM in HFMD treatment is still insufficient to determine the efficacy, due to the numerous factors, such as the quality control of CM [164, 165], and sufficient sample sizes, improved randomization, and better group organization in clinical studies.

## Conclusion

This review summarized the group of anti-EV71 molecules that have been isolated from CHM and have been applied clinically for this purpose.

## Abbreviations

EV71: enterovirus 71
HFMD: hand, foot and mouth disease
IFNs: interferons
CPE: cytopathic effect
HRV: human rhinovirus
CHM: Chinese herbal medicine
CM: Chinese medicine
SI: selective index
HBV: Hepatitis B virus
CVB3: Coxsackievirus B3
HSV: Herpes simplex virus
CVA16: Coxsackievirus A16
IL: interleukin
KGS: *Kalanchoe gracilis*
EA: acetate
BuOH: *n*-butanol
SMGGT: Sheng-Ma-Ge-Gen-Tang
RSV: respiratory syncytial virus
GQP: GuiQi polysaccharides
IRES: internal ribosome entry site
PI4KB: phosphatidylinositol 4-kinase IIIβ
OSBP: Oxysterol-binding protein
CR: chrysin
CPI: chrysin phosphate ester
ROS: reactive oxygen species
EGCG: Epigallocatechin gallate
GCG: Gallocatechin gallate
GLTA: Lanosta-7,9(11),24-trien-3-one,15; 26-dihydroxy
GLTB: Ganoderic acid Y
MLB: Magnesium lithospermate B
RA: Rosmarinic acid
HIV: human immunodeficiency virus
